# HepaCAM associates with connexin 43 and enhances its localization in cellular junctions

**DOI:** 10.1038/srep36218

**Published:** 2016-11-07

**Authors:** Meihui Wu, Mei Chung Moh, Herbert Schwarz

**Affiliations:** 1 Department of Physiology, Yong Loo Lin School of Medicine, National University of Singapore, Singapore 117597; 2 Immunology Programme, Life Sciences Institute, National University of Singapore, Singapore 117456

## Abstract

HepaCAM (GlialCAM) is frequently deleted in carcinomas, and reintroduction of hepaCAM into transformed cell lines reduces cellular growth and induces senescence. Mutations in *HEPACAM* give rise to the neurodegenerative disease megalencephalic leukoencephalopathy with subcortical cysts (MLC) since mutated hepaCAM prevents shuttling of MLC1 protein to astrocytic junctions in the plasma membrane. Here we identify that hepaCAM associates with connexin 43, a main component of gap junctions, and enhances connexin 43 localization to the plasma membrane at cellular junctions. HepaCAM also increases the levels of connexin 43, not by enhancing its transcription but by stabilizing connexin 43 protein. In the absence of hepaCAM, connexin 43 undergoes a faster degradation via the lysosomal pathway while proteasomal degradation seems not to be involved. Mutations in hepaCAM that cause MLC, or neutralization of hepaCAM by antibodies disrupt its association with connexin 43 at cellular junctions. By discovering the requirement of hepaCAM for localizing connexin 43, a well-established tumor suppressor, to cellular junctions and stabilizing it there, this study suggests a mechanism by which deletion of hepaCAM may support tumor progression.

Cell adhesion molecules (CAMs) are cell surface proteins that mediate cell-extracellular matrix (ECM) and cell-cell interactions. These molecules with tightly-regulated expression are essential for the development and maintenance of tissue architecture. Besides mediating cell adhesion, there is increasing evidence that CAMs also function as receptors which modulate signal transduction in many cellular processes including proliferation, apoptosis, migration and differentiation. Deregulation of these biological processes in malignant tumors has been associated with the aberrant expression of CAMs, demonstrating that alterations in CAMs play a pivotal role in cancer development and progression^1^,^2^.

HepaCAM was first identified as a cell adhesion molecule which is frequently downregulated in hepatocellular carcinoma^3^. HepaCAM is a member of the immunoglobulin superfamily and consists of an extracellular domain with two immunoglobulin loops, a transmembrane segment and a cytoplasmic tail^4^.

HepaCAM has been found to be downregulated in hepatocellular carcinoma, and reexpression of hepaCAM in hepaCAM-negative hepatocellular carcinoma cells inhibits their growth^3^ which is characteristic of a tumor suppressor. Similar data have been obtained for many other solid cancers. HepaCAM is suppressed in carcinomas of the breast, kidney, colon, rectum and stomach^5^. HepaCAM reexpressed in renal carcinoma and breast cancer cells inhibited cell proliferation and colony formation, and induced cell senescence^5^,^6^,^7^,^8^. In bladder cancer, it has been found that the expression of hepaCAM is silenced by hypermethylation, and reversal of hypermethylation by inhibiting DNA methyltransferases led to the reexpression of hepaCAM and reduction in cell growth^9^,^10^,^11^. Further, hepaCAM expression has been shown to induce differentiation of glioblastoma cells^12^. In addition, hepaCAM regulates cell adhesion and migration^3^,^4^,^13^,^14^, processes which are essential for normal development and metastasis.

HepaCAM was also discovered in the central nervous system (CNS) where it was called GlialCAM^15^. HepaCAM associates with MLC1, and is required to shuttle MLC1 to the cell membrane where it localizes to cellular junctions. Mutations in either gene, *HEPACAM* or *MLC1*, lead to the development of the neurodegenerative disease megalencephalic leukoencephalopathy with subcortical cysts (MLC)^16^. HepaCAM also associates with the chloride channel ClC-2^17^.

In this study we demonstrate that hepaCAM associates with the major gap junction protein connexin 43 and shuttles it to cellular junctions on the cell surface. Further, hepaCAM stabilizes connexin 43 protein at cellular junctions. Antagonistic anti-hepaCAM antibodies and hepaCAM mutations that cause MLC also prevent its association with connexin 43. Since connexin 43 has anti-tumor activity, its regulation by hepaCAM may explain the anti-tumor activity of hepaCAM.

## Results

### HepaCAM associates with connexin 43 and regulates its localization and expression

**Figure 1:**
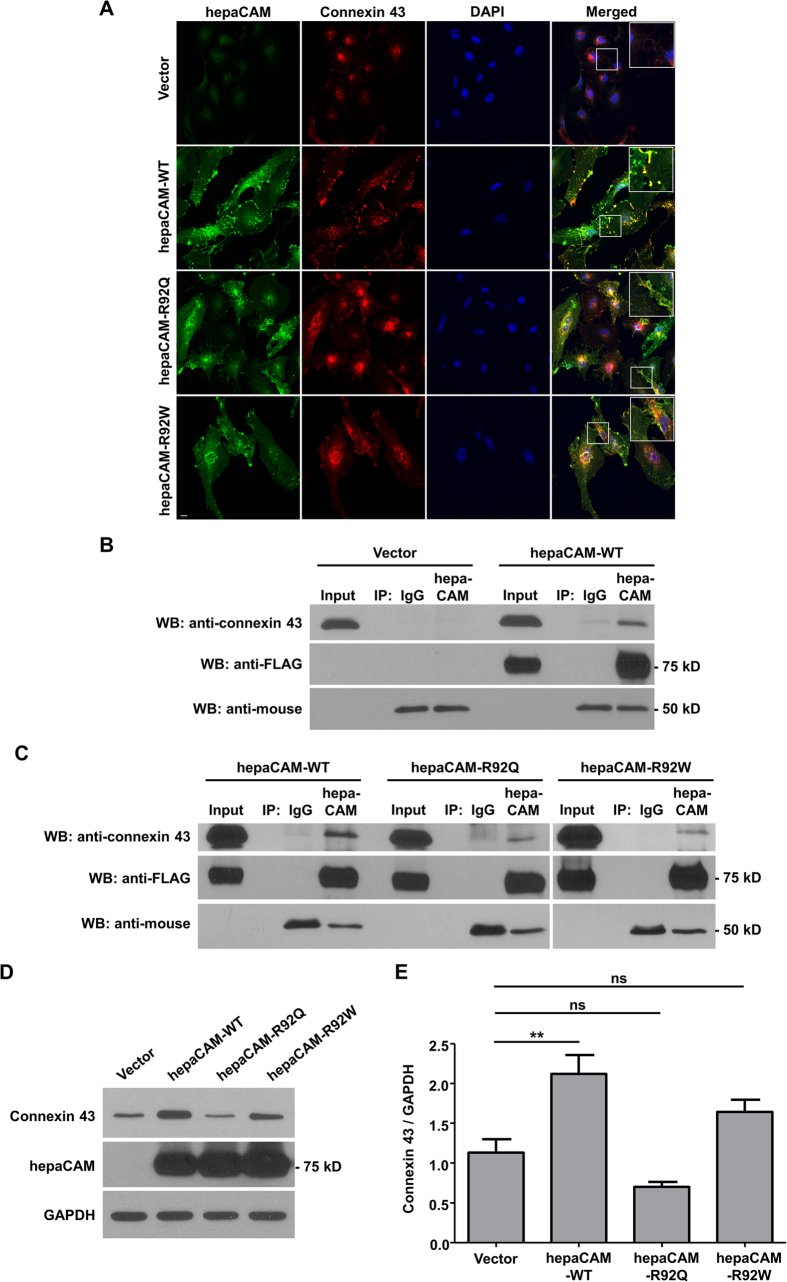
HepaCAM associates with connexin 43. (**A**) U373 MG cells were stably transfected with pcDNA3.1 vector, wild-type hepaCAM, hepaCAM-R92Q and hepaCAM-R92W. Immunofluorescent staining was performed with antibodies against the hepaCAM cytoplasmic domain (green) and connexin 43 (red). Co-localization of hepaCAM and connexin 43 is indicated by yellow fluorescence. Nuclei were stained with DAPI (blue). Insets show a higher magnification of sites of cell-cell contacts. Cells were visualized by confocal microscopy under a 60× objective. Scale bar: 10 μm. (**B**) Co-immunoprecipitatation of connexin 43 and hepaCAM. Cell lysates were prepared from U373 MG cells stably transfected with pcDNA3.1 vector and wild-type hepaCAM, and immunoprecipitated with antibody against the hepaCAM extracellular domain (IP hepaCAM). Immunoprecipitation with mouse IgG1 (IP IgG) was included as a negative control. Western blot analysis was performed on the immunoprecipitates and input (3%) using connexin 43 antibody. The efficiency of hepaCAM immunoprecipitation was evaluated with an HRP-conjugated FLAG antibody. The IgG heavy chain detected with an HRP-conjugated anti-mouse antibody is shown as a loading control. (**C**) Co-immunoprecipitation of wild-type and mutant hepaCAM with connexin 43. Cell lysates were immunoprecipitated with antibody against the hepaCAM extracellular domain (IP hepaCAM). Immunoprecipitation with mouse IgG1 (IP IgG) was included as a negative control. Western blot analysis was performed on the immunoprecipitates and input (2%) using connexin 43 antibody. (**D**) Expression of wild-type hepaCAM increases connexin 43 protein levels in U373 MG cells. 20 μg of cell lysates were subjected to Western blot analysis. GAPDH was used as a loading control. The result presented is a representative experiment of four independent experiments with similar results. The full view blots are shown in Supplementary Figure 1. (**E**) Quantification of connexin 43 protein levels in D and in three additional independent Western blot analyses. Using ImageJ the densities of the connexin 43 bands were normalized to the densities of the respective GAPDH bands for each sample, and the mean relative density over the four experiments was calculated. The data presented are the means ± SE (n = 4), **p < 0.01 as assessed by one-way ANOVA with Tukey’s multiple comparison test.

Since hepaCAM has been shown to associate with MLC1, a gap junction protein^16^, we hypothesized that hepaCAM may also associate with connexin 43 a component of gap junctions. Indeed, the two proteins colocalized at cell-cell contacts of U373 MG cells as shown by confocal microscopy (Fig. 1A). Connexin 43 is predominantly localized intracellularly in U373 MG cells. Expression of WT hepaCAM redistributed connexin 43 to the cell surface, particularly to sites of cell-cell contacts, where colocalization of the two molecules was detected (Fig. 1A). Since mutations in hepaCAM can cause the disease MLC, we also investigated the effects of mutations on connexin 43 localization. We selected two naturally occurring mutations, R92Q and R92W, in the hepaCAM extracellular domain, specifically in the first Ig-like domain. These two mutations not only cause MLC but also diminished the colocalization with connexin 43 at cell-cell contacts (Fig. 1A).

The physical interaction of WT hepaCAM and connexin 43 was verified by a co-immunoprecipitation assay (Fig. 1B). The R92Q and R92W mutations, however, diminished the physical association of hepaCAM and connexin 43 since the two proteins could hardly be co-precipitated when carrying one of the two mutations (Fig. 1C).

The confocal staining of connexin 43 in the hepaCAM-transfected cells indicated not only a cellular redistribution of connexin 43 by hepaCAM but also an induction of connexin 43 expression (Fig. 1A). An enhancement of connexin 43 expression by WT hepaCAM was confirmed by Western blot analysis (Fig. 1D,E). However, the two hepaCAM mutations, especially R92Q, were less effective in enhancing connexin 43 expression (Fig. 1D,E).

**Figure 2:**
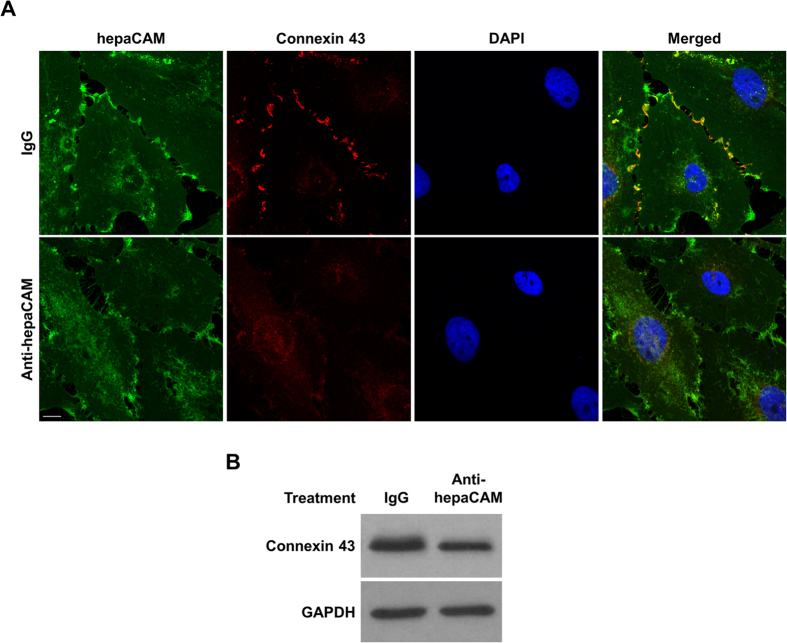
HepaCAM enhances connexin 43 expression. (**A**) Treatment of hepaCAM-expressing U373 MG cells with antibodies against the hepaCAM extracellular domain prevents the association of hepaCAM with connexin 43 at cell-cell contacts. Wild-type hepaCAM-expressing U373 MG cells were treated overnight with antibody against the hepaCAM extracellular domain in soluble form (10 μg/ml). Cells were also treated with the isotype mouse IgG1 as a control. The next day, cells were fixed and immunofluorescent staining was performed with antibodies against the hepaCAM extracellular domain (green) and connexin 43 (red). Co-localization of hepaCAM and connexin 43 is indicated by yellow fluorescence. Nuclei were stained with DAPI (blue). Cells were visualized by confocal microscopy under a 60× objective. Scale bar: 10 μm. (**B**) Treatment of hepaCAM-expressing U373 MG cells with antibodies against the hepaCAM extracellular domain causes a downregulation of connexin 43 expression. Wild-type hepaCAM-expressing U373 MG cells were treated overnight with antibody against the hepaCAM extracellular domain in soluble form (10 μg/ml). Cells were also treated with the isotype mouse IgG1 as a control. The next day, cells were lysed and 20 μg of cell lysates were subjected to Western blot analysis using connexin 43 antibody. GAPDH was used as a loading control. The full view blots are shown in Supplementary Figure 2.

Similar to the mutations in hepaCAM, neutralization of hepaCAM by an antibody diminished its colocalization with connexin 43 at cell-cell contacts (Fig. 2A). In addition, anti-hepaCAM antibody treatment resulted in decreased connexin 43 protein levels (Fig. 2B). The reduction in connexin 43 levels in U373 MG cells by neutralization of hepaCAM appears to be stronger in the confocal images compared to the Western blot. This is possibly due to lesser connexin 43 clustering at the cell membrane in the presence of the anti-hepaCAM antibody, which affects the intensity of the confocal staining more than the signal on the Western blot. Wang and Rose^18^ had observed that a high expression of connexin 43 did not necessarily result in immunostaining for connexin 43 clusters at cell-cell contacts.

### HepaCAM stabilizes connexin 43

**Figure 3:**
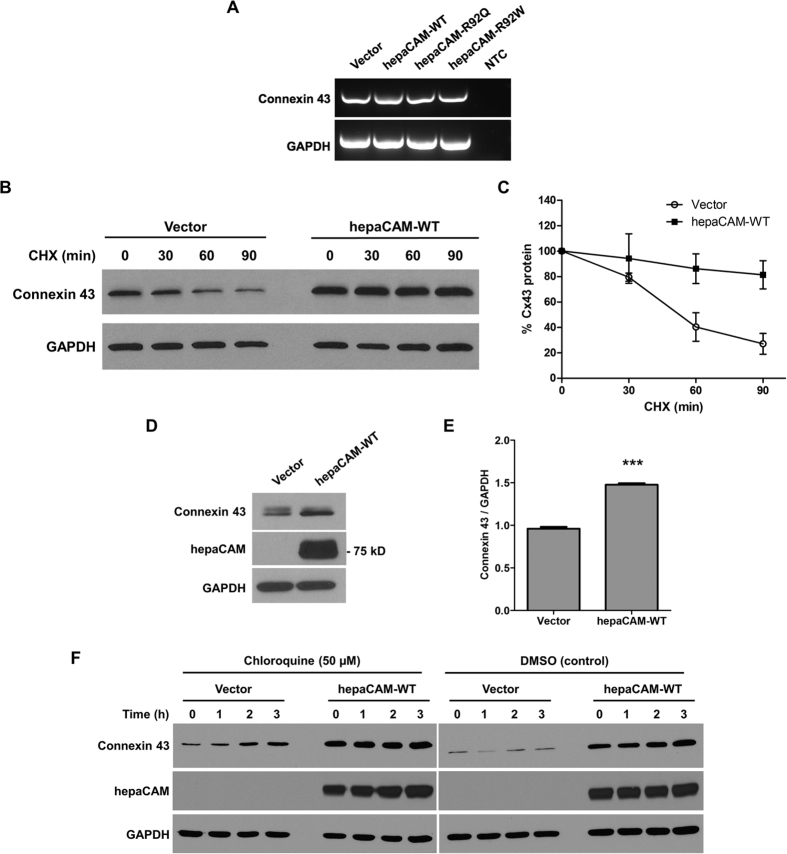
HepaCAM regulates connexin 43 stability. (**A**) Evaluation of connexin 43 mRNA expression. Total RNA was analyzed by RT-PCR. GAPDH and no template controls (NTC) were included as housekeeping gene and negative controls, respectively. (**B**) Evaluation of connexin 43 protein stability by a cycloheximide (CHX) chase assay. Cells treated with CHX (50 μg/ml) for the times indicated were lysed and 30 μg of cell lysates were subjected to Western blot analysis. The result presented is a representative experiment of three independent experiments with similar results. (**C**) Quantification of all three CHX chase experiments using ImageJ. The densities of the connexin 43 bands were normalized to the densities of the respective GAPDH bands at each time-point. The level of connexin 43 remaining at each time-point was calculated as a percentage of the initial connexin 43 level (time 0 of CHX treatment). The data presented are the means ± SE (*n* = 3). (**D**) Expression of hepaCAM in HEK293T cells increases connexin 43 protein levels. HEK293T cells were transiently transfected with pcDNA3.1 vector or wild-type hepaCAM. Two days after transfection, cells were lysed and 60 μg of cell lysates were subjected to Western blot analysis using antibodies against connexin 43 and the hepaCAM extracellular domain. The result presented is a representative experiment of three independent experiments with similar results. (**E**) Quantification of all three experiments using ImageJ. The densities of the connexin 43 bands were normalized to the densities of the respective GAPDH bands for each sample, and the mean relative density over the three experiments was calculated. The data presented are the means ± SE (n = 3), ***p < 0.0001 as assessed by *t*-test. (**F**) HepaCAM slows down connexin 43 turnover by the lysosomal pathway. U373 MG cells stably transfected with pcDNA3.1 vector or wild-type hepaCAM were treated with chloroquine (50 μM) and 30 μg of cell lysates were subjected to Western blot analysis for connexin 43. The result presented is representative of two independent experiments with similar results. The full view blots for (**B**,**D**,**F**) are shown in Supplementary Figures 3,4 and 5, respectively.

The reduced connexin 43 levels in the absence of hepaCAM is consistent with the higher connexin 43 levels in cells expressing WT compared to mutated hepaCAM (Fig. 1D). However, the presence of hepaCAM did not enhance transcription of connexin 43 since connexin 43 mRNA levels did not differ between cells expressing no hepaCAM or WT or mutated hepaCAM (Fig. 3A), which suggests that hepaCAM stabilizes connexin 43 protein, or that hepaCAM induces a translation-mediated or posttranslational increase in connexin 43. To further assess whether hepaCAM influences connexin 43 protein stability, the kinetics of connexin 43 degradation were determined by a cycloheximide (CHX) chase experiment. Cells expressing WT hepaCAM had increased stability of connexin 43, as shown by its slower rate of degradation upon inhibition of translational elongation with CHX (Fig. 3B,C). The influence of hepaCAM on connexin 43 stability was supported by observations of enhanced connexin 43 protein levels in HEK293T cells transiently transfected with WT hepaCAM (Fig. 3D,E). Treatment with the lysosome inhibitor chloroquine led to a gradual increase in connexin 43 expression in control U373 MG cells (Fig. 3F), while treatment with the proteasome inhibitor MG132 had no effects (data not shown). This suggests that connexin 43 is predominantly degraded by the lysosomal pathway in U373 MG cells, and that hepaCAM expression slows down connexin 43 turnover via this pathway.

## Discussion

HepaCAM has been identified as a tumor suppressor^3^. But the underlying mechanism(s) of hepaCAM-mediated suppression of cancer cell growth is largely unknown. A gene expression analysis of hepaCAM-expressing and control bladder cancer cells identified a large number of differentially regulated genes, indicating that hepaCAM may mediate its growth-inhibitory effect via multiple pathways^19^.

Connexin 43, like hepaCAM, has been identified as a tumor suppressor protein^20^,^21^. Both molecules are frequently downregulated in cancers and exert growth-inhibitory activities. In addition, several studies have also shown an abnormal localization of connexin 43 in the cytoplasm of tumor cells, instead of the cell membrane^22^. As with previous studies^23^, a predominant localization of connexin 43 in the cytoplasm of glioblastoma cells was observed in this study. Since hepaCAM increases connexin 43 levels, and promotes its trafficking from the cytoplasm to cellular junctions, the deletion of either gene may have similar growth-promoting effects for cancer cells.

**Figure 4:**
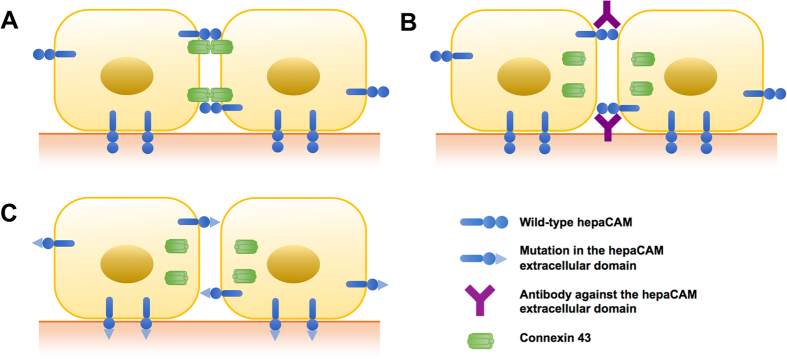
Schematic depiction of hepaCAM activities. (**A**) HepaCAM associates with connexin 43 at the cell-cell contacts of U373 MG glioblastoma cells. (**B**) Treatment of cells with antibody against the HepaCAM extracellular domain prevents association of hepaCAM with connexin 43 at cell-cell contacts and downregulates cell surface expression of connexin 43. (**C**) The R92Q and R92W mutations in the hepaCAM extracellular domain prevent association of hepaCAM with connexin 43 at cell-cell contacts.

In the absence of hepaCAM, connexin 43 may become unstable or may cease to get shuttled from the cytoplasm to the plasma membrane, and therefore its amount gradually diminishes at cellular junctions, which may lead to reduced cell-cell communication (Fig. 4A,B). Connexin 43 is known to undergo rapid turnover^24^, either by the proteasomal and/or the lysosomal pathways^25^. We found connexin 43 expression to be increased in U373 MG cells expressing WT hepaCAM, which is not due to an increase in connexin 43 transcription, but due to a slower rate of turnover by the lysosomal pathway. The increased stability of connexin 43 protein depends on its interaction with hepaCAM, as treatment of cells with an antibody against the hepaCAM extracellular domain disrupts this interaction and causes a downregulation in connexin 43 expression. Similarly, the R92Q and R92W mutations in hepaCAM, which lead to a weaker interaction with connexin 43, did not enhance connexin 43 protein levels in U373 MG cells, unlike WT hepaCAM. This is in line with previous observations that the interaction of hepaCAM with MLC1 in rat astrocytes increases the protein stability of MLC1^26^.

Neutralization of hepaCAM by antibodies (Fig. 4B) or inactivation by mutations (Fig. 4C) reduced not only expression of connexin 43 but also its localization at the plasma membrane. This is paralleled by the finding that hepaCAM ablation reduced expression of MLC1 at the plasma membrane^26^. Both MLC1 and connexin 43 are structural components of cellular junctions which implies that hepaCAM may be a general regulator of cellular junctions. Similarly, hepaCAM associates with the chloride channel ClC-2, and coexpression of hepaCAM with ClC-2 in HeLa cells led to an increased presence of ClC-2 on the plasma membrane^17^.

It had been found that it is the extracellular domain of hepaCAM that is necessary for its targeting to cell junctions, and for its interactions with itself and with MLC1 and ClC-2^27^. This finding is in line with our data that the mutations as well as the monoclonal antibody that interrupt the association of hepaCAM with connexin 43 are located in or target the extracellular domain.

By discovering the requirement of hepaCAM for localizing connexin 43, a well-established tumor suppressor, to cellular junctions and stabilizing it there, this study identifies a mechanism by which deletion of hepaCAM may support tumor progression.

## Materials and Methods

### Cell culture and transfection

U373 MG glioblastoma cells (a kind gift from Dr. Celestial Yap, National University of Singapore) were maintained in F-12 medium (Life Technologies, Carlsbad, CA, USA) supplemented with 10% FBS (Biowest, Nuaille, France). Wild-type hepaCAM/GlialCAM (WT), GlialCAM-R92Q, and GlialCAM-R92W were cloned into the pcDNA3.1 vector (Life Technologies) 5′ to a FLAG epitope. Stable transfection of U373 MG cells was performed using Lipofectamine PLUS reagent (Life Technologies) according to the manufacturer’s instructions. Cells were selected in culture medium containing 800 μg/ml G418 (Sigma-Aldrich, St. Louis, MO, USA) for 2 weeks before cloning. Human embryonic kidney HEK293T cells (a kind gift from Dr. Paul MacAry, National University of Singapore) were maintained in in high glucose Dulbecco’s Modified Eagle Medium (DMEM; Sigma-Aldrich) supplemented with 10% FBS. Transient transfection of HEK293T cells was performed using Turbofect Transfection Reagent (Thermo Scientific, Rockford, IL, USA) according to the manufacturer’s instructions.

### Treatment of cells with hepaCAM antibody

U373 MG cells stably expressing hepaCAM were treated overnight with a monoclonal antibody against the extracellular hepaCAM domain (clone 419305; R&D Systems, Minneapolis, MN, USA) at a concentration of 10 μg/ml. Mouse IgG1 (MOPC-21; Sigma-Aldrich) was used as an isotype control.

### Immunofluorescence

Cells were cultured on glass coverslips to 80% confluency, fixed with 4% paraformaldehyde and permeabilized with 0.2% Triton X-100. Non-specific sites were blocked with 1% BSA. Subcellular localization of hepaCAM was detected with a custom-made mouse monoclonal antibody against the cytoplasmic hepaCAM domain (GenScript, Piscataway, NJ, USA) or with an antibody against the extracellular hepaCAM domain (clone 419305), followed by Alexa Fluor 488 goat anti-mouse IgG (Life Technologies). Subcellullar localization of connexin 43 was detected with rabbit polyclonal anti-connexin 43 (clone 3512; Cell Signaling Technology, Danvers, MA, USA) and Alexa Fluor 594 goat anti-rabbit IgG (Life Technologies). Cells were counterstained with DAPI, and the coverslips were mounted onto glass slides for viewing with an Olympus FV1000 confocal laser scanning microscope.

### Western blot analysis and co-immunoprecipitation

Cells were lysed in radioimmunoprecipitation assay (RIPA) buffer supplemented with a protease inhibitor cocktail (Sigma-Aldrich). For co-immunoprecipitation experiments, cells were lysed in lysis buffer (1% NP-40 in PBS) supplemented with protease inhibitors. The pre-cleared cell lysates (1 mg protein) were incubated with mouse anti-hepaCAM (clone 419305) or mouse IgG1 (MOPC-21) and protein G agarose beads (Thermo Scientific) overnight at 4 °C. The immunoprecipitates were washed four times with 1% NP-40 lysis buffer, re-suspended in Laemmli sample buffer and boiled for 10 min to elute the bound proteins. Protein samples were resolved by SDS-PAGE and transferred to PVDF membranes. Connexin 43 expression was detected with rabbit anti-connexin 43 (clone 3512). Expression of hepaCAM was detected with antibody against the extracellular hepaCAM domain (clone 419305) or with mouse anti-FLAG-horseradish peroxidase (HRP) antibody (clone 5A8E5; Genscript), where indicated. Mouse anti-GAPDH (Santa Cruz Biotechnology, Dallas, TX, USA) was used as a loading control. Bound unconjugated primary antibodies were detected with the appropriate HRP-conjugated secondary antibodies (Santa Cruz Biotechnology) and visualized by enhanced chemiluminescence (Thermo Scientific).

### RNA isolation and RT-PCR

Total RNA was isolated with the RNeasy Mini Kit (Qiagen, Hilden, Germany) and subjected to on-column DNase digestion (Qiagen) to eliminate trace genomic DNA contamination. Semi-quantitative RT-PCR to determine connexin 43 mRNA expression was performed with the OneStep RT-PCR Kit (Qiagen). The primers for connexin 43 were 5′-GGG TTA AGG GAA AGA GCG ACC-3′ (sense) and 5′-CCC CAT TCG ATT TTG TTC TGC-3′ (antisense), as described previously by Eugenin *et al*.^28^. GAPDH was included as a housekeeping gene control. Reverse transcription of the RNA (0.5 μg) was carried out at 50 °C for 30 min, followed by incubation at 95 °C for 15 min to activate the HotStarTaq DNA polymerase. The PCR conditions used for the cDNA amplification were 94 °C for 30 s, 50 °C for 30 s, 72 °C for 1 min, for a total of 34 cycles. The RT-PCR products were subsequently resolved in 1.5% agarose gels.

### Cycloheximide chase assay and treatment with chloroquine

To determine the stability of connexin 43 protein, cells were treated with 50 μg/ml cycloheximide (C4859; Sigma-Aldrich), a potent inhibitor of protein synthesis. At each time-point, cells were lysed with RIPA buffer supplemented with protease inhibitors. Cell lysates were analysed by Western blot and quantification of the bands was performed using the ImageJ software (National Institutes of Health, Bethesda, Maryland, USA).

To determine if connexin 43 is degraded by the lysosomal pathway, cells were treated with 50 μM of the lysosome inhibitor chloroquine (C6628; Sigma-Aldrich) and lysed with RIPA buffer supplemented with protease inhibitors at each time-point.

### Statistical analysis

Statistical significance was determined by a two-tailed unpaired Student’s *t*-test, or one-way ANOVA. Values of *p* < 0.05 were considered significant.

## Additional Information

**How to cite this article**: Wu, M. *et al*. HepaCAM associates with connexin 43 and enhances its localization in cellular junctions. *Sci. Rep.* **6**, 36218; doi: 10.1038/srep36218 (2016).

**Publisher’s note**: Springer Nature remains neutral with regard to jurisdictional claims in published maps and institutional affiliations.

## Supplementary Material

Supplementary Information
