# Supplementary material for: HepaCAM associates with connexin 43 and enhances its localization in cellular junctions
Source: Sci Rep. 2016 Nov 7;6:36218. doi: 10.1038/srep36218 (PMC5098153; doi:10.1038/srep36218)
Supplement: Supplementary Information [file srep36218-s1.doc]

**HepaCAM associates with connexin 43 and enhances its localization in cellular junctions**

**Meihui Wu^1,2^, Mei Chung Moh^1,2^ and Herbert Schwarz^1,2^**

^1^Department of Physiology, ^2^Immunology Programme, Life Sciences Institute, National University of Singapore, Singapore. Correspondence and requests for materials should be addressed to H.S. (email: [phssh@nus.edu.sg](mailto:phssh@nus.edu.sg)).

Running title: HepaCAM associates with connexin 43

Key words: HepaCAM, connexin 43, tumor suppression

**
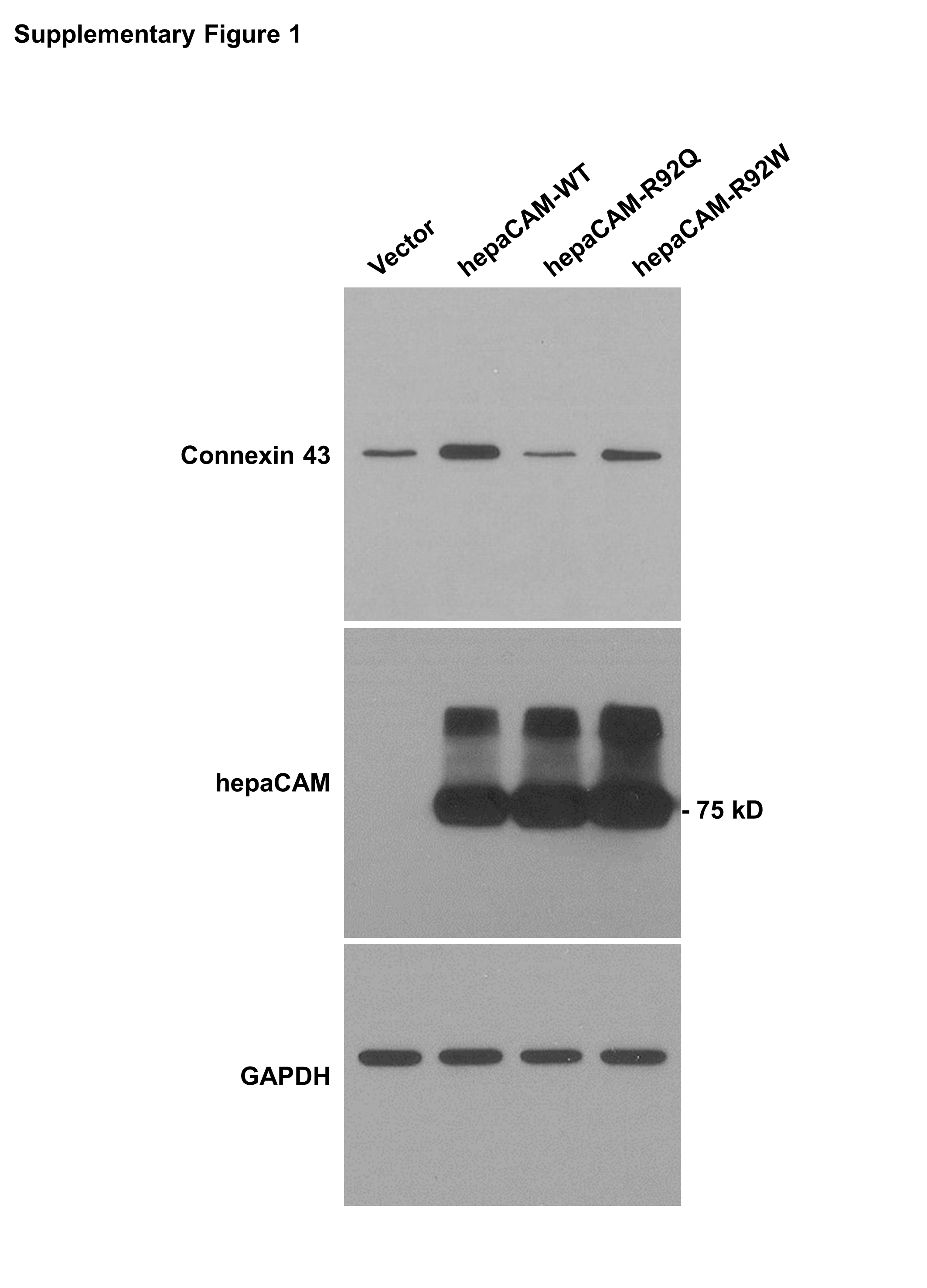
**

**Supplementary Figure 1. Whole view of Western blot for Figure 1D.** Expression of wild-type hepaCAM increases connexin 43 protein levels in U373 MG cells. 20 μg of cell lysates were subjected to Western blot analysis. GAPDH was used as a loading control. The result presented is a representative experiment of four independent experiments with similar results.

**
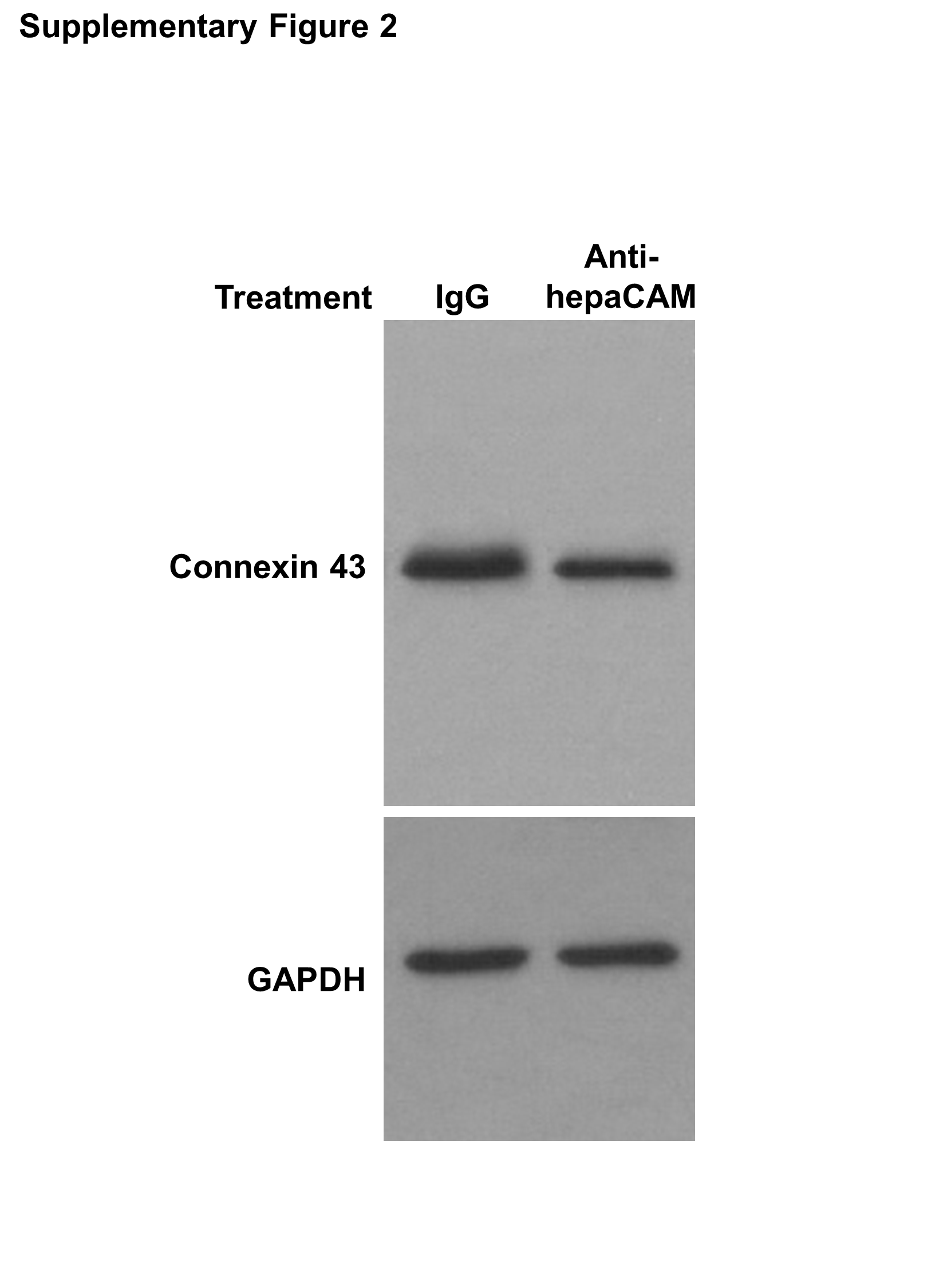
**

**Supplementary Figure 2. Whole view of Western blot for Figure 2B.** Treatment of hepaCAM-expressing U373 MG cells with antibodies against the hepaCAM extracellular domain causes a downregulation of connexin 43 expression. Wild‑type hepaCAM-expressing U373 MG cells were treated overnight with antibody against the hepaCAM extracellular domain in soluble form (10 μg/ml). Cells were also treated with the isotype mouse IgG1 as a control. The next day, cells were lysed and 20 μg of cell lysates were subjected to Western blot analysis using connexin 43 antibody. GAPDH was used as a loading control.

**
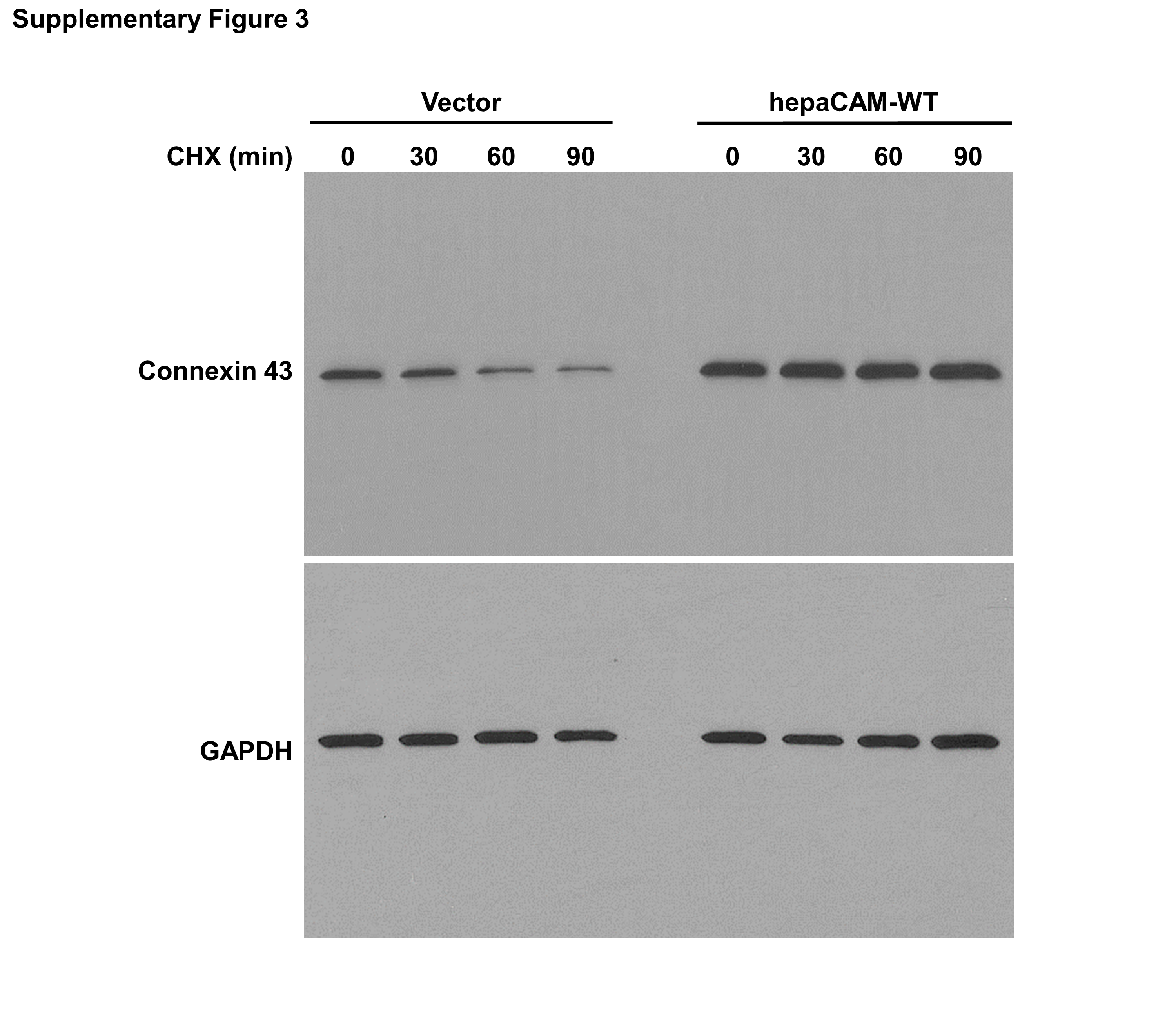
**

**Supplementary Figure 3. Whole view of Western blot for Figure 3B.** Evaluation of connexin 43 protein stability by a cycloheximide (CHX) chase assay. Cells treated with CHX (50 μg/ml) for the times indicated were lysed and 30 μg of cell lysates were subjected to Western blot analysis. The result presented is a representative experiment of three independent experiments with similar results.

**
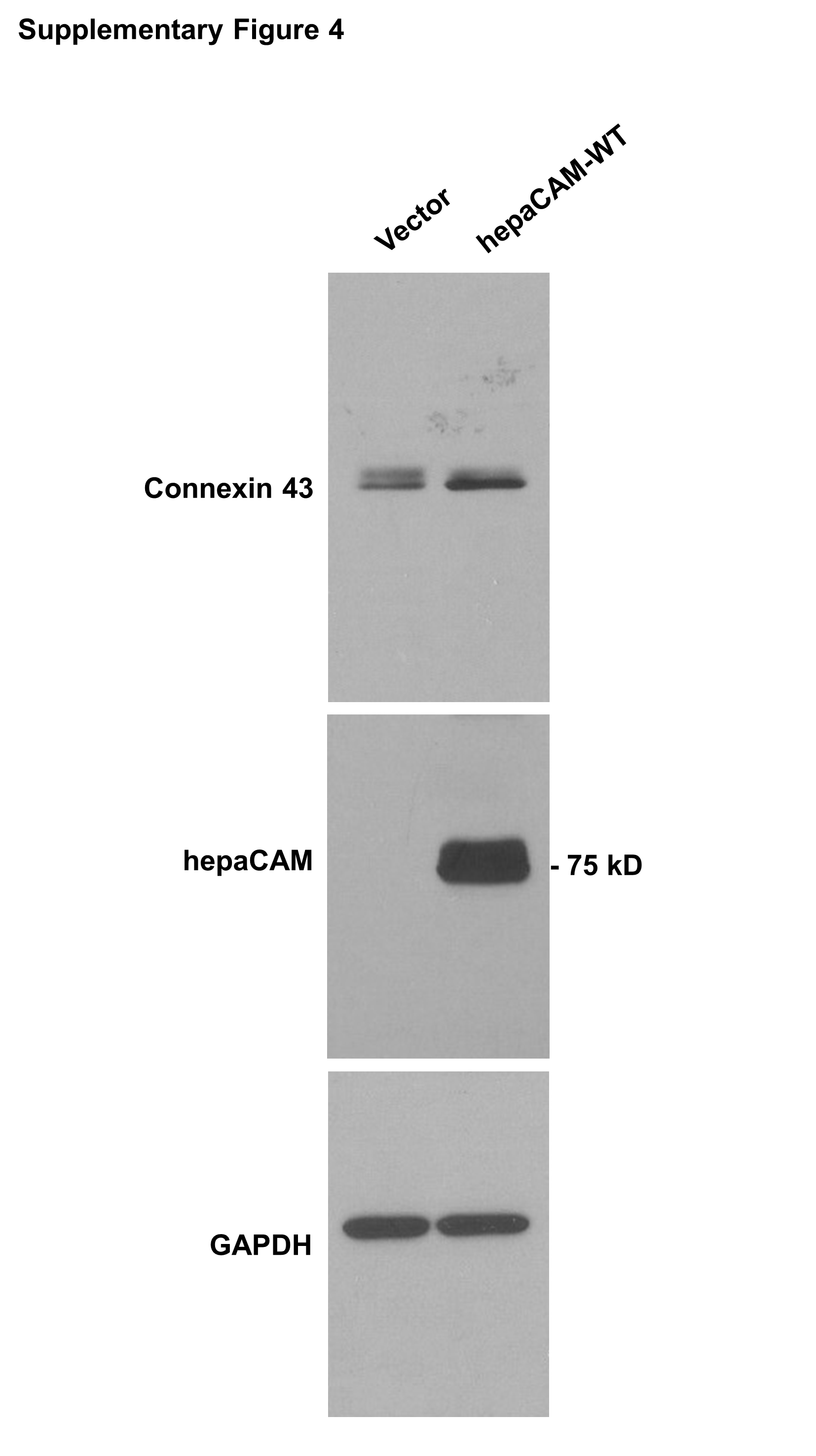
**

**Supplementary Figure 4. Whole view of Western blot for Figure 3D.** Expression of hepaCAM in HEK293T cells increases connexin 43 protein levels. HEK293T cells were transiently transfected with pcDNA3.1 vector or wild-type hepaCAM. Two days after transfection, cells were lysed and 60 μg of cell lysates were subjected to Western blot analysis using antibodies against connexin 43 and the hepaCAM extracellular domain. The result presented is a representative experiment of three independent experiments with similar results.

**
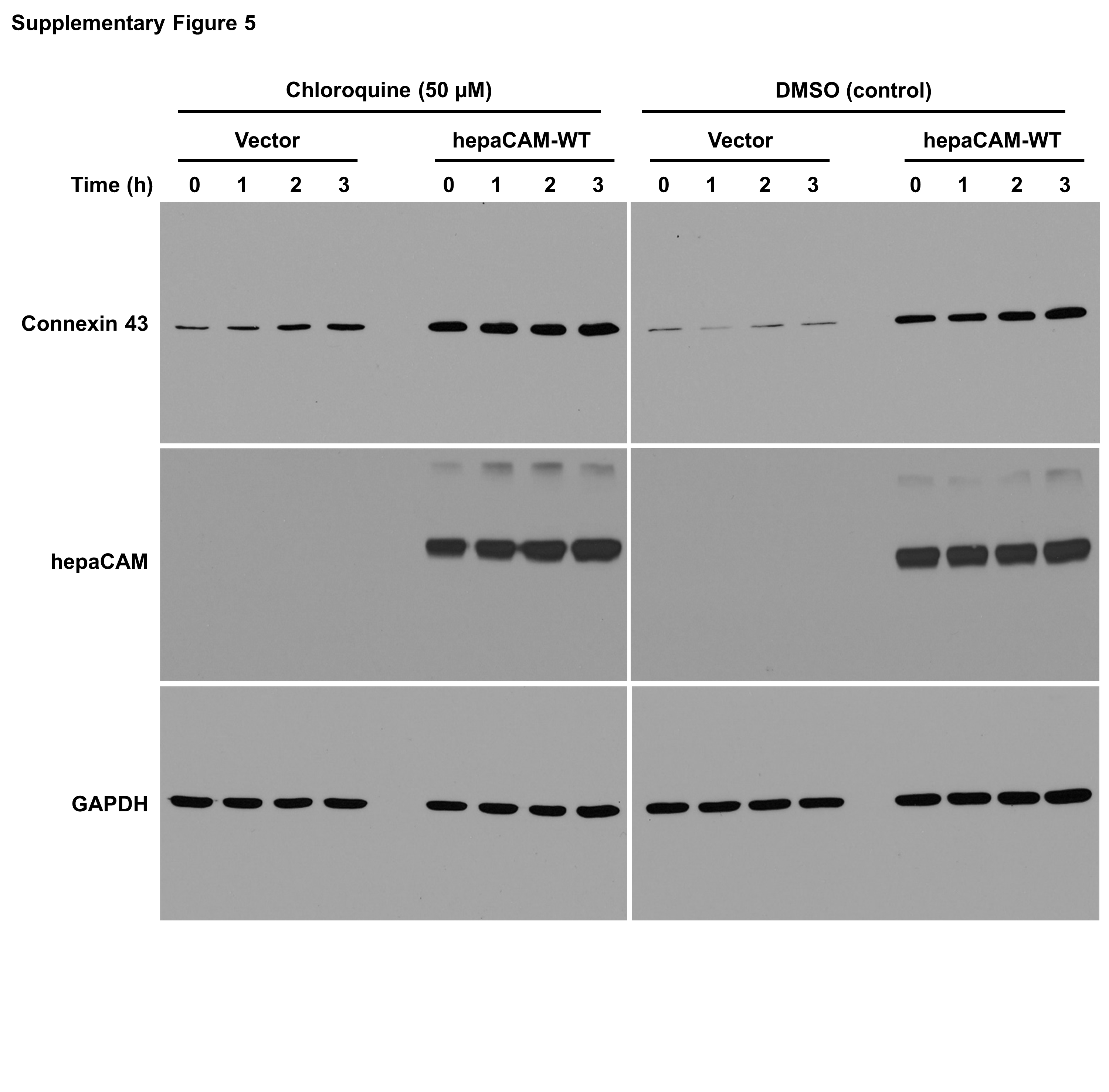
**

**Supplementary Figure 5. Whole view of Western blot for Figure 3F.** hepaCAM slows down connexin 43 turnover by the lysosomal pathway. U373 MG cells stably transfected with pcDNA3.1 vector or wild-type hepaCAM were treated with chloroquine (50 μM) and 30 μg of cell lysates were subjected to Western blot analysis for connexin 43. The result presented is representative of two independent experiments with similar results.
